# Nutrition Literacy Status and Preferred Nutrition Communication Channels Among Adults in the Lower Mississippi Delta

**Published:** 2009-09-15

**Authors:** Jamie Zoellner, Carol Connell, Wendy Bounds, LaShaundrea Crook, Kathy Yadrick

**Affiliations:** Virginia Polytechnic Institute and State University, Human Nutrition Foods and Exercise. At the time of the research described in this article, Dr Zoellner was affiliated with the University of Southern Mississippi, Hattiesburg, Mississippi; University of Southern Mississippi, Hattiesburg, Mississippi; University of Southern Mississippi, Hattiesburg, Mississippi; University of Southern Mississippi, Hattiesburg, Mississippi; University of Southern Mississippi, Hattiesburg, Mississippi

## Abstract

**Introduction:**

The objective of this cross-sectional study was to examine the nutrition literacy status of adults in the Lower Mississippi Delta.

**Methods:**

Survey instruments included the Newest Vital Sign and an adapted version of the Health Information National Trends Survey. A proportional quota sampling plan was used to represent educational achievement of residents in the Delta region. Participants included 177 adults, primarily African Americans (81%). Descriptive statistics, χ^2 ^analysis, analysis of variance, and multivariate analysis of covariance tests were used to examine survey data.

**Results:**

Results indicated that 24% of participants had a high likelihood of limited nutrition literacy, 28% had a possibility of limited nutrition literacy, and 48% had adequate nutrition literacy. Controlling for income and education level, the multivariate analysis of covariance models revealed that nutrition literacy was significantly associated with media use for general purposes (*F* = 2.79, *P* = .005), media use for nutrition information (*F* = 2.30, *P* = .04), and level of trust from nutrition sources (*F* = 2.29, *P* = .005). Overall, the Internet was the least trusted and least used source for nutrition information. Only 12% of participants correctly identified the 2005 MyPyramid graphic, and the majority (78%) rated their dietary knowledge as poor or fair.

**Conclusion:**

Compared with other national surveys, rates of limited health literacy among Delta adults were high. Nutrition literacy status has implications for how people seek nutrition information and how much they trust it. Understanding the causes and consequences of limited nutrition literacy may be a step toward reducing the burden of nutrition-related chronic diseases among disadvantaged rural communities.

## Introduction

The continuing increases in rates of nutrition-related chronic diseases suggest that many Americans lack basic health literacy and nutrition literacy skills. Without such skills, people cannot access and understand public health information such as that in the *2005 Dietary Guidelines for Americans *(Dietary Guidelines) (1) and MyPyramid Food Guidance System (http://www.mypyramid.gov/).

Nutrition literacy may be defined as the degree to which people have the capacity to obtain, process, and understand basic nutrition information. Nutrition literacy is vital to residents of places with education, health, and nutrition disparities, such as the Lower Mississippi Delta. The Delta region is predominantly rural and has a high concentration of African Americans, high rates of poverty, and low educational achievement. Residents in the Delta have a disproportionately high prevalence of chronic diseases, including obesity, heart disease, diabetes, and hypertension, and in general have poorer adherence to dietary recommendations than the US population (2-5). Although these disparities are well documented, no known published research has examined the health or nutrition literacy of residents in the Delta region.

The goal of this cross-sectional study was to explore nutrition literacy among adults in the Delta region. Because the Dietary Guidelines, MyPyramid, and Nutrition Facts Panel (http://www.fda.gov/Food/LabelingNutrition/ConsumerInformation/default.htm) are the cornerstones to adopting nutrition recommendations, these resources were integral to our study. We investigated the associations between nutrition literacy and 1) the use of media channels, 2) level of trust in nutrition information sources, 3) confidence in getting information about nutrition, and 4) barriers to seeking nutrition information, while accounting for potential confounding variables.

## Methods

### Survey instruments

To describe the capacity to obtain basic nutrition information, we developed 4 questions to understand awareness of and exposure to the Dietary Guidelines and MyPyramid. In addition, 43 questions from the Health Communication section of the National Cancer Institute Health Information National Trends Survey 2 (HINTS 2; http://cancercontrol.cancer.gov/hints/) were adapted to assess exposure to nutrition and health information (6). HINTS 2 was originally developed to understand how adults use different communication channels to obtain health information and has been widely used to characterize cancer knowledge and awareness, trusted sources of cancer information, and preferences for cancer information (7-11). For this research, a notable adaptation to HINTS 2 was revising references to "cancer" or "health" to "nutrition, food, or diet." Content of the questions was validated by a 4-member expert panel (1 doctoral-level health communication researcher and 3 doctoral-level registered dietitians). The expert panel gave feedback on the survey's content, clarity, and cognitive complexity. The instrument then underwent 2 rounds of cognitive interviewing with 9 participants by using concurrent, structured verbal probing techniques (12). After appropriate changes were made, the instrument was pilot tested in a sample of 21 Delta residents, by using retrospective, structured verbal probing techniques (12). This pilot testing resulted in minor changes to the wording of a few questions.

The capacity to understand nutrition information was measured by using the previously developed and validated Newest Vital Sign (NVS) (13). The NVS involves having patients view information on a nutrition information label and then answer 6 questions about how they would interpret and act on the information contained on the label. The number of correct responses is summed to produce a nutrition literacy score ranging from 0 to 6. Zero or 1 correct answers indicates a high likelihood of limited literacy, 2 to 3 correct answers indicates the possibility of limited literacy, and virtually all participants with scores of 4 to 6 have adequate literacy skills. The NVS has been validated against the Test of Functional Health Literacy in Adults (TOFHLA) in 500 English-speaking and Spanish-speaking primary care patients residing in Arizona (13).

### Data collection

This research was approved by the University of Southern Mississippi's institutional review board. Community health advisors as research partners (CHARPs) were trained to recruit participants from their communities according to the sampling plan and to collect data. CHARPs are community members who have completed training on cancer awareness provided by the Deep South Network for Cancer Control (a National Cancer Institute-funded project) and who have successfully helped recruit subjects or collect data for several research projects in the Delta (14,15). For this nutrition literacy research, the CHARPs were required to attend a 2-day training session. On the second day, each CHARP was required to pass a certification session in which the investigators observed them completing a survey with a mock participant. Five CHARPs completed the training, passed the certification, and collected data for the study. The investigators continually monitored data quality throughout the study. Data were collected at locations convenient to the participants, including the participant's home or office, the CHARP's home, libraries, and community centers. Participants were given a $25 gift card.

The target population for this cross-sectional study was adults residing in 6 Mississippi Delta counties. In the context of this health literacy research, we sought an accurate representation of education levels in these 6 counties to ensure that the results were generalizable. Therefore, a proportional quota sampling plan based on the 2000 US Census Data education levels was used (http://www.census.gov/). Education achievement data for the 6 counties were averaged to determine percentage of the population estimated in 6 education strata (Table 1).

To simplify the sampling plan matrix for the CHARPs, sex, race, and other demographic characteristics were not directly accounted for or required in the sampling and recruiting plan. However, the CHARPs were trained on the need for a representative sample, educated on the proportional demographics of the region, and encouraged to recruit an equal number of men and women and approximately 70% African American and 30% white participants. On the basis of the power analysis for an *F* test (analysis of variance) with 3 nutrition literacy groups, 150 participants would provide sufficient power (80% at α = .05) to detect a moderate effect size (f = 0.25) (G*Power 3.0.8 [Heinrich-Heine-Universität, Düsseldorf, Germany]). A plan to survey 180 respondents was then developed to account for potential incomplete data sets and loss of data, and to allow for some logistical flexibility in the sampling plan among CHARPs. Data were collected during November 2006-April 2007.

### Data analysis

Descriptive statistics including means, standard deviations, and frequencies were used to summarize all responses. The associations of demographic characteristics (sex, race, age, income level, and education level) with survey responses were evaluated by using χ^2^ and 1-way analysis of variance tests. Because nutrition literacy scores varied significantly by income and educational level, these covariates were controlled for in multivariate analysis of covariance tests using nutrition literacy category as the independent variable and survey responses as the dependent variables. As a follow-up to the multivariate analysis of covariance models, pairwise comparisons using univariate F tests were used to evaluate differences among nutrition literacy categories. When appropriate, χ^2^ and univariate tests were used to examine the relationships between nutrition literacy and survey responses. Significance is reported at *P* < .05. All statistical analyses were performed by using SPSS version 15.0 (SPSS, Inc, Chicago, Illinois).

## Results

Most participants were African American (81%) and female (70%) (Table 1). The proportional quota sampling plan was sufficiently achieved. Furthermore, the distribution of age ranges was well represented. Body mass index (BMI), calculated using self-reported height and weight, revealed that 82% of the participants were categorized as overweight or obese. Nutrition literacy scores varied significantly by income level and educational achievement but not by race, sex, age, or BMI (Table 1).

When categorizing nutrition literacy according to NVS scoring procedures, scores indicated that 42 (24%) participants had a high likelihood of limited literacy skills (0-1 correct answers), 50 (28%) had a possibility of limited literacy skills (2-3 correct answers), and 85 (48%) had adequate literacy skills (4-6 correct answers). Several significant differences were revealed when examining the relationships between nutrition literacy categories and participants' use of communication channels both for general purposes and for obtaining information related to nutrition, food, or diet (Table 2). When general use of media channels was examined, 27.8 hours per week (standard deviation [SD] 16.5 h/wk) were spent viewing television, which was nearly twice as high as the 15.6 (15.2) hours per week spent listening to the radio and more than 4 times higher than the 6.5 (9.9) hours per week spent on the Internet. On average, participants reported reading the newspaper 2.9 (2.5) days per week. Controlling for income and education level, nutrition literacy was associated with use of these media channels (*F* = 2.79, *P* = .05). The follow-up pairwise comparisons revealed that only television viewing varied significantly among groups; participants in the lowest nutrition literacy category reported significantly more hours of television viewing for general purposes than did the other 2 groups.

Subsequently, participants were asked to report which media channels they had used in the past 12 months to obtain nutrition, food, or diet information. Overall, the most frequently confirmed media channel for nutrition information was television (57%), followed by newspapers or magazines (50%). Only 20% confirmed using the Internet to obtain nutrition information. We found a significant positive linear association between using a media channel and nutrition literacy; as literacy increased, the proportion of participants using a channel increased. When respondents were asked to report frequency of media use for nutrition information, television was used the most overall at 1.9 (SD = 2.4) times per month, followed by newspapers or magazines at 1.4 (SD = 2.1) times per month, and then the Internet at 0.5 (SD = 1.5) times per month. Nutrition literacy category was associated with frequency of media use for nutrition information (*F* = 2.30, *P* = .035). The follow-up pairwise comparisons revealed that participants with lower literacy skills used television and newspapers or magazines less frequently than did those with adequate literacy skills. When examining demographic effects on use of media channels, the only significant (*P* < .001) difference was that adults aged 61 years or older used the Internet less frequently than did all other age groups.

Overall, participants trusted information from doctors or health care providers and television the most and from the Internet the least (Table 3). People in the lowest nutrition literacy category had lower trust in magazines, newspapers, and radio than did those with adequate nutrition literacy skills (*F* = 2.29, *P* = .05). However, no trust differences were found among nutrition literacy categories for trust in health care providers, television, family or friends, and the Internet. Although people with lower literacy skills had less confidence in obtaining nutrition information, this trend did not achieve significance (*F* = 2.64, *P* = .07). Overall ratings for barriers to seeking nutrition information were relatively neutral (neither agree nor disagree), and the multivariate analysis of covariance model for barriers was not significant (*F *= 0.84, *P* = .57).

When respondents were asked if they were aware that the government had released new dietary guidelines in 2005, 76% of the participants indicated they were not aware. When asked to identify the most recent picture promoted by the dietary guidelines, only 22 (12%) correctly identified the MyPyramid graphic. Most participants (46%) selected the 1994 Food Guide Pyramid graphic, followed by the Four Basic Food Groups graphic (23%), and Canadian Food Guide graphic (9%). When asked to rate their knowledge of the Dietary Guidelines on a 5-point Likert scale (1 = poor, 5 = very good), the average was 1.8 (1.0); most perceived their knowledge as poor (53%) or fair (25%). Cumulatively, only 7% of participants perceived their knowledge to be good or very good. None of these survey responses differed by demographic characteristics. However, participants with adequate literacy scores rated their knowledge of the Dietary Guidelines higher at 2.0 (1.0) compared with those who had a possibility of limited literacy skills at 1.5 (0.9) and those with a high likelihood of limited literacy at 1.6 (1.0) (*P* = .02). Of the 22 respondents who correctly identified the MyPyramid graphic, 13 had adequate nutrition literacy, 6 had possibility of limited nutrition literacy, and 3 had high likelihood of limited nutrition literacy.

## Discussion

Although educational and health disparities in the Delta region are well documented, no other published studies have directly examined the health or nutrition literacy status of residents (5,6,16). The finding that most (52%) participants had a high likelihood or a possibility of limited literacy skills helps establish the scope of health literacy among adults in the Delta region. The proportional sampling of educational achievement and adequate distribution of ages provides reasonable assurance that these nutrition literacy findings are generalizable to the greater Delta region. Although *Healthy People 2010* established the objective to improve the health literacy of people with inadequate or marginal literacy skills, this is a developmental objective; therefore, baseline data and targets have not been established (17).

The National Assessment of Adult Literacy (NAAL) recently released the first large-scale study of health literacy among approximately 19,000 US adults (18). The comprehensive assessment examined prose, document, and quantitative health literacy for 3 domains of health and health care information and services: clinical, prevention, and navigation of the health system. Analyses were weighted to represent the total US population. Results indicate that 12% of US adults have proficient health literacy, 53% have intermediate health literacy, 22% have basic health literacy, and 14% have below-basic health literacy. Because of methodologic differences in assessing and scoring health literacy, a precise comparison between the NAAL health literacy findings and our findings is difficult (16,18). However, crude comparisons of these national data to our data from the rural Mississippi Delta suggest that health literacy rates in the Delta may differ from those of the general US population. These suggested differences call for further exploration. The NAAL study revealed that health literacy increases with each higher level of educational attainment and that people living below the poverty level have lower average health literacy than do those above it. Our findings, which identify significant relationships between educational achievement and nutrition literacy scores and between income level and nutrition literacy scores, support the NAAL findings. Although our study did not identify race, age, or sex differences between nutrition literacy categories, the NAAL study indicated that blacks have lower average health literacy than whites, adults aged 65 or older have lower average health literacy than younger age groups, and the average health literacy scores for men are lower than those for women (18).

In our study, we assessed nutrition information-seeking behaviors and defined seeking as an active and purposeful effort to obtain nutrition information. Our results suggest a clear association between nutrition-seeking behaviors and nutrition literacy. The significant linear-by-linear association with nutrition literacy category and each media source we queried, including television, newspapers/magazines, and the Internet, indicates that nutrition information-seeking increases as nutrition literacy skills increase. Other researchers have studied cancer-related information-seeking behaviors and distinguish seeking behaviors from scanning behaviors, where scanning is defined as passive or casual exposure to information (19,20). Scanning for and seeking cancer-related information are unmistakably separate behaviors that have clear associations with sociodemographic characteristics, lifestyle behaviors, cancer knowledge, and several health-relevant outcomes such as fruit and vegetable intake (21,22). However, a limitation of our study is that we were unable to specifically distinguish between nutrition information-scanning and information-seeking behaviors. The differences between nutrition information-scanning and information-seeking behaviors and their relationships to nutrition literacy and dietary behaviors warrant further investigation.

The low use of the Internet for general purposes and for seeking information related to nutrition, food, or diet was a finding of this study. The Internet was also the least trusted source of nutrition information. With launch of the www.MyPyramid.gov Web site, the Internet appears to be the major communication channel used to promote the 2005 Dietary Guidelines and MyPyramid key messages. During the past decade, the Internet has caused a nationwide revolution in health information access, and in national surveys the Internet is consistently ranked among the most popular sources of health information (10). However, our findings suggest that the Internet is not a frequently used or trusted source of nutrition information among adults in the Delta region. Not only is television viewing more than 4 times higher than Internet use, television is also a more trusted source of nutrition information. These findings suggest that television is a more appropriate media channel for disseminating health and nutrition information for this population and imply a need to increase the number of scientifically based messages related to dietary recommendations provided during television programming. Although trust of nonprint sources (including doctors or other health care providers, television, and family or friends) did not vary among literacy categories, people with lower literacy rated their trust in print sources (including magazines and newspapers) lower than did those in higher nutrition literacy categories. We also noted that people with lower nutrition literacy reported less confidence in getting advice or information about nutrition and rated barriers to seeking nutrition information as higher than did those with adequate literacy. However, the trend was not significant after accounting for covariates. These results identify associations between seeking nutrition information and nutrition literacy. Although the NAAL study did not assess trust, barriers, or confidence in seeking health information, the results indicated that, compared with adults who had higher health literacy, those with lower health literacy receive less information about health from written sources, including the Internet (18).

This research was conducted between November 2006 and April 2007, approximately 2 years after release of the Dietary Guidelines in January of 2005 and MyPyramid in April of 2005. Only 12% of the Delta residents surveyed could correctly identify the MyPyramid graphic, and most respondents were not aware of the new *2005 Dietary Guidelines* and rated their knowledge as poor. These findings may not be comparable to those for other populations; no other published research has examined the degree to which these new recommendations have reached other populations. Nevertheless, this finding illustrates poor dissemination of nutrition recommendations to this rural region of the Delta, where health disparities are common.

The fact that 82% of participants in this study were classified as overweight or obese, compared with a national average of 66%, illustrates the nutrition- and obesity-related health disparities experienced by this Delta population (21). Furthermore, considering that people tend to underreport weight, the documented rates of overweight and obesity based on self-reported measures in this study may be understated (22).

This study is not without limitations. The primary limitation is that temporality cannot be determined in this cross-sectional design. Furthermore, potential limitations are also imposed by the survey instruments. Validation of the NVS was conducted in a primary care setting where only 5% of the participants were African American (16). Therefore, use of NVS to assess literacy levels in a community-based setting with mostly African Americans should be accounted for in the interpretation of this study. Although appropriate efforts were taken to establish content and face validity of the modified HINTS instrument, this is the lowest level of validity and also imposes study limitations. Finally, no questions were targeted at exploring access to the Internet. The proportion of participants who had access to the Internet should be assessed and accounted for in future research.

Notwithstanding these limitations, our findings have several implications for practice and policy. First, if awareness of and access to trusted nutrition information is problematic, the likelihood of adopting healthy nutrition recommendations is greatly diminished. If health and nutrition professionals expect to compete with nutrition claims made through television and other types of advertising, they must understand and use appropriate communication channels and overcome barriers to nutrition information use. Second, interpretations of our findings suggest it may be unrealistic to expect people with low nutrition literacy to seek information, regardless of the source. The problem of low nutrition literacy is then partially shifted to nutrition educators to develop and deliver targeted nutrition outreach interventions that deemphasize the use of printed materials and remove the burden on people to seek nutrition information on their own. The complexity of health literacy is affected not only by individual skills but also by the organizations responsible for the delivery of health information and services. Finally, the link between health literacy and disease prevention and health promotion has not been fully explored because most research on health literacy has focused on the health care setting (23-31). Because health literacy in the context of primary prevention can affect public health, our study emphasizes the need to understand limited health and nutrition literacy in nonprimary care settings.

These results suggest that the use of technology for health communication is problematic for impoverished rural areas. Understanding the causes and consequences of limited nutrition literacy may help effectively communicate science-based nutrition information and reduce the burden of nutrition-related chronic diseases among members of disadvantaged rural communities. Future studies are needed to 1) evaluate the validity of health and nutrition literacy screening instruments for African American populations in nonprimary care settings, 2) explore the effect of relying on the Internet as a central mode of health communication in impoverished rural regions, and 3) determine if focused attention on nutrition literacy is an effective intervention strategy for reducing the burden of obesity and other nutrition-related chronic diseases among disadvantaged populations with health disparities.

## Figures and Tables

**Table 1:** Characteristics and Nutrition Literacy Among Adults (N = 177) in the Lower Mississippi Delta, 2006-2007

Characteristics	No. (%)	**Nutrition Literacy Score,** a Mean (SD)	***P* Value** b
**Race**			
African American	144 (81)	3.12 (1.96)	.21
White	33 (19)	3.61 (2.15)	
**Sex**			
Female	124 (70)	3.27 (2.00)	.51
Male	53 (30)	3.06 (2.02)	
**Age, y^c^**			
18-30	31 (18)	3.16 (1.88)	.16
31-40	29 (16)	3.62 (2.15)	
41-50	42 (24)	3.21 (1.95)	
51-60	39 (22)	3.46 (1.79)	
≥61	33 (19)	2.45 (2.22)	
**Annual income, $**			
<5,000	19 (11)	1.84 (2.04)	<.001
5,000-14,999	52 (29)	2.63 (2.08)	
15,000-24,999	29 (16)	2.93 (1.71)	
25,000-34,999	24 (14)	3.50 (1.69)	
35,000-44,999	20 (11)	3.70 (2.03)	
≥45,000	16 (9)	5.31 (0.87)	
Don't know/refused	17 (10)	4.00 (1.37)	
**Highest level of education completed**			
Less than 9th grade	28 (16)	2.43 (1.69)	.008
9th to 12th grade, some high school	41 (23)	2.88 (1.99)	
High school diploma or GED	37 (21)	2.92 (2.18)	
Some college or specialized training, no degree	36 (20)	3.81 (1.85)	
Associate's or bachelor's degree	22 (13)	3.59 (1.97)	
Attended graduate school	13 (7)	4.46 (1.76)	
**Body mass index^c^,^d^,** **kg/m^2^**			
Underweight (<18.5)	0	NA	.85
Healthy weight (18.5-24.9)	31 (18)	3.16 (2.21)	
Overweight (25.0-29.9)	55 (31)	3.11 (1.97)	
Obese (≥30.0)	90 (51)	3.30 (1.97)	

Abbreviations: SD, standard deviation; GED, general equivalency diploma; NA, not applicable.

a Assessed using the Newest Vital Sign (13) with scores ranging from 0 to 6: 0 or 1 correct answers, high likelihood of limited literacy; 2-3 correct answers, possibility of limited literacy; 4-6, adequate literacy skills.

b One-way analysis of variance for difference in nutrition literacy score among demographic variables.

c The sample size does not equal 177 because of missing responses.

d Calculated by using self-reported height and weight.

**Table 2:** Use of Media Channels for General Purposes and for Seeking Information About Nutrition Among Adults in the Lower Mississippi Delta, 2006-2007

Media Use	Overall(N = 177)	Nutrition Literacy Category			*P* Value

		Category 1: High Likelihood of Limited Literacy(n = 42)	Category 2: Possibility of Limited Literacy(n = 50)	Category 3: Adequate Literacy(n = 85)	
**Frequency of media use for general purposes, mean (SD)^a^**					
Television, h/wk^b^	27.8 (16.5)	35.9 (15.9)	25.7 (13.0)	25.1 (17.5)	<.001^c^
Radio, h/wk	15.6 (15.2)	17.4 (14.5)	14.1 (14.5)	15.6 (15.9)	.45^c^
Internet, h/wk	6.5 (9.9)	5.4 (11.0)	5.5 (9.7)	7.7 (9.4)	.88^c^
Newspaper, d/wk	2.9 (2.5)	2.6 (2.5)	2.3 (2.2)	3.4 (2.6)	.41^c^
**Media use for seeking information about nutrition, food, or diet in the past 12 months, no. (%)**					
Confirmed using television for nutrition information	101 (57)	17 (40)	20 (40)	64 (75)	.001^d^
Confirmed using newspaper or magazine for nutrition information	88 (50)	15 (36)	18 (36)	55 (65)	<.001^d^
Confirmed using Internet for nutrition information	36 (20)	4 (10)	7 (14)	25 (29)	.008^d^
**Frequency of media use for information about nutrition, food, or diet, mean (SD)^e^**					
Television for nutrition information, no. of times per month^f^	1.9 (2.4)	1.5 (2.5)	1.2 (2.0)	2.6 (2.5)	.04^c^
Newspaper or magazine for nutrition information, no. of times per month^g^	1.4 (2.1)	0.6 (0.9)	1.3 (2.1)	1.9 (2.3)	.02^c^
Internet for nutrition information, no. of times per month	0.5 (1.5)	0.5 (1.8)	0.2 (0.3)	0.7 (1.8)	.43^c^

a Multivariate analysis of covariance main effect of media use for general purposes (*F* = 2.79, *P* = .05); controlled for income and educational level.

b In a pairwise comparison of adjusted means, category 1 > category 2 and category 3.

c Univariate *F* test.

d Mantel-Haenszel χ^2^ (linear-by-linear association); pairwise comparison does not apply.

e Multivariate analysis of covariance main effect of media use for information about nutrition (*F* = 2.30, *P* = .04); controlled for income and educational level.

f In a pairwise comparison of adjusted means, category 3 > category 2.

g In a pairwise comparison of adjusted means, category 3 > category 1.

**Table 3:** Trust, Confidence, and Barriers to Seeking Nutrition Information Among Adults in the Lower Mississippi Delta (N = 177), 2006-2007

Nutrition-Seeking Behavior	OverallMean (SD)	Nutrition Literacy Category			*P* Value

		Category 1: High Likelihood of Limited Literacy,Mean (SD)	Category 2: Possibility of Limited Literacy,Mean (SD)	Category 3: Adequate Literacy,Mean (SD)	
**Level of trust of nutrition, food, or diet information sources^a^**					
Doctor or other health care provider	3.6 (0.7)	3.5 (0.6)	3.5 (0.8)	3.7 (0.6)	.57
Television	3.0 (0.7)	2.9 (0.9)	3.0 (0.7)	3.0 (0.7)	.89
Family or friend	2.8 (0.7)	2.7 (0.8)	2.8 (0.7)	2.8 (0.7)	.94
Magazine^b^	2.7 (0.8)	2.5 (0.8)	2.4 (0.9)	3.0 (0.6)	.008
Newspaper^b^	2.6 (0.9)	2.2 (0.8)	2.5 (1.0)	2.9 (0.7)	.004
Radio^b^	2.5 (0.8)	2.8 (0.9)	2.4 (0.8)	2.7 (0.7)	.008
Internet	2.3 (1.0)	1.9 (1.0)	2.2 (1.0)	2.2 (1.0)	.09
**Confidence in getting information about nutrition, food, or diet^c^**					
Confidence	3.6 (0.9)	3.3 (1.2)	3.6 (0.9)	3.8 (0.8)	.07
**Barriers to seeking information about nutrition, food, or diet^d^**					
It took a lot of effort to get the information you needed	3.1 (1.4)	3.6 (1.4)	3.5 (1.4)	2.7 (1.3)	.10
You felt frustrated during your search	2.7 (1.4)	3.1 (1.7)	3.1 (1.5)	2.3 (1.1)	.13
You were concerned about the quality	3.5 (1.5)	3.5 (1.4)	3.0 (1.5)	3.5 (1.5)	.78
The information you found was too hard to understand	2.6 (1.3)	3.0 (1.6)	2.7 (1.4)	2.4 (1.2)	.44

a Overall (n = 176), category 1 (n = 41), category 2 (n = 50), category 3 (n = 85). Means are reported on a 4-point scale of 1) not at all, 2) a little, 3) some, and 4) a lot. Multivariate analysis of covariance main effect trust (*F* = 2.29, *P* = .005); controlled for income and educational level.

b In a pairwise comparison of adjusted means, category 3 > category 1.

c Overall (n = 174), category 1 (n = 40), category 2 (n = 50), category 3 (n = 84). Means are reported on a 5-point scale of 1) not at all confident, 2) a little confident, 3) somewhat confident, 4) very confident, and 5) completely confident. Univariate main effect confidence (*F* = 2.64, *P* = .07); controlled for income and educational level.

d Overall (n = 103), category 1 (n = 19), category 2 (n = 27), category 3 (n = 57). Reduced numbers are due to skip pattern in questionnaire. Means are reported on a 5-point scale of 1) strongly disagree, 2) somewhat disagree, 3) neither agree or disagree, 4) somewhat agree, and 5) strongly agree. Multivariate analysis of covariance main effect barriers (*F* = 0.84, *P* = .57); controlled for income and educational level.
